# Public engagement by early career researchers in East Africa during the COVID-19 pandemic: case studies from East Africa

**DOI:** 10.12688/openresafrica.13897.1

**Published:** 2023-03-20

**Authors:** Trizah K. Milugo, Mary V. Mosha, Eddie Wampande, Rune Philemon, Immaculate N. Lwanga, Janet Seeley, Nelson K. Sewankambo

**Affiliations:** 1 International Centre of Insect Physiology and Ecology, Nairobi, Kenya; 2 Technical University of Kenya, Nairobi, Kenya; 3 Institute of Public Health, Kilimanjaro Christian Medical University College, Moshi, Tanzania; 4 Makerere University, College of Veterinary Medicine, Animal Resources and Bio Security, Kampala, Uganda; 5 Makerere University College of Health Sciences, Makerere, Uganda; 6 London School of Hygiene and Tropical Medicine,, London, UK

**Keywords:** East Africa, COVID-19, Community engagement, Pandemic

## Abstract

**Background:** Community engagement and involvement (CEI) in research usually depends on face-to-face interactions. However, the COVID-19 pandemic prevented such interactions because of national lockdowns and social distancing. This paper highlights the ways in which early career researchers from East Africa tackled CEI activities during the pandemic.

**Methods:** We provide four case examples that illustrate how early-career researchers based in Kenya, Uganda and Tanzania, deployed different approaches and initiatives to community-engaged research during the pandemic to encourage participation and uptake of research findings.

**Results:** All the three early-career researchers attempted to use virtual/digital means to implement the CEI. However, in each country, this attempt was unsuccessful because of poor connectivity, as well as many poorer students lacking access to telephones and computers. Nevertheless, the researchers effectively engaged the students using different activities (making up songs, drawing comics, and taking part in quizzes) once the schools reopened.

**Conclusion:** These results highlight the complexity of implementing community engagement and involvement in health research when face-to-face interaction is not possible. The findings are relevant to researchers who wish to incorporate community engagement in their research and initiatives.

**Keywords:** East Africa, COVID-19, Community engagement, Pandemic

## Introduction

Community engagement and involvement (CEI) in research is increasingly seen as important in ensuring that those who take part in research not only have information on the study but can also take part in decision making about the research, including the interpretation and dissemination of results ( Holzer *et al.*, 2014; Tembo *et al.*, 2021; Tindana *et al.*, 2007). As Kroese *et al.* (2021) note, 'meaningful CEI within research is important as it reduces the potential for exploitation of communities and facilitates the implementation of health research' (p. 2). In recent years, partly in response to donors encouraging the greater inclusion of CEI activities in research funding applications, guidelines and recommendations have been published setting out ways in which science communication can be enhanced to increase public engagement ( Akukwe, 1999; World Health Organization, 2017). Humm and Schrögel (2020), for example, recommend seven ways to reach `underserved and marginalised communities with science communication' (p.6). These are: `listening to underserved audiences, reducing the distance, illustrating the relevance of science for daily life, going where the people are, cooperating with stakeholders, and multipliers, as well as [recognising] the problem of too much openness [providing more information than people can absorb at once]. And [rather than] one-time activities [encouraging long-term activities]' (p. 1).

The COVID-19 pandemic posed an immediate challenge to CEI based on such guidance. The widespread use of lockdowns to limit the spread of the virus prevented researchers involved in research, of any sort, from going where the people they were working with were located and thus `reducing distance'. Different ways of engaging and maintaining contact with research participants have thus been needed ( Manikam *et al.*, 2021; Tindana *et al.*, 2020). In response, there has been a plethora of publications in the past two years describing the public engagement approaches used to research different populations with COVID-19 messaging, including those related to clinical and vaccine research (see for example, Afolabi & Ilesanmi, 2021; Gilmore *et al.*, 2022; Ratneswaren, 2020). There are also findings emerging on the experience of engaging the public in research on topics other than COVID-19 during the pandemic. We contribute to this second body of work by describing the experiences of early career researchers in East Africa during the pandemic as they modified their CEI plans and approaches for their health-related doctoral and post-doctoral studies. It is anticipated that these findings will inform future research endeavours in the area of community engagement and involvement in health research.

Our colleagues have described elsewhere the community engagement efforts of three early career researchers in Kenya, Tanzania and Uganda funded under the Training Health Researchers into Vocational Excellence in East Africa (THRiVE) project ( Bargul *et al.*, 2022). That project was a collaborative research capacity building project involving five universities (Makerere University, Gulu University, Kilimanjaro Christian Medical University College, University of Cambridge, London School of Hygiene and Tropical Medicine); and three research institutes (Uganda Virus Research Institute, International Centre for Insect Physiology and Ecology in Kenya and the National Institute of Medical Research, in Mwanza-Tanzania) which aimed to develop world-class researchers and research leaders who could conduct high-quality independent research and transform communities where they live and work. As part of that effort, the researchers were supported to develop their skills in CEI. In this paper, we focus specifically on the ways in which community engagement efforts had to change during the pandemic as the researchers pivoted their approach in response to non-pharmacological responses to the spread of the virus, such as national lockdowns and social distancing.

We begin by describing the measures imposed in Kenya, Tanzania, and Uganda to prevent the spread of COVID-19. We then provide some background on the THRiVE project, and the four researchers and their research funded through THRiVE before describing their plans for community engagement activities before the pandemic and then the changes they made as the COVID-19 pandemic spread in East Africa. We conclude by reflecting on the lessons we have learnt from this experience.

## The measures imposed in each country to prevent the spread of COVID-19 which affected research activities

In Kenya and Uganda, the first cases of COVID-19 were reported in March of 2020. That month, a total lockdown was put in place in both countries. In Uganda a full lockdown, restricting movement outside the home and a night curfew, lasted until early May. While this first lockdown was subsequently lifted, advice on physical distancing, frequent handwashing and the use of facemasks was retained. However, all schools were closed for 22 months, up until the beginning of 2022. In Kenya, a nationwide dawn-to-dusk curfew was put in place, and movement into and out of urban areas with high transmission rate of COVID-19 restricted. This was implemented in an effort to reduce mass migration to rural areas where at-risk populations, especially elderly persons, reside ( Wangari *et al.,* 2021). Learning institutions were also closed, whilst workplaces were advised to allow individuals to work from home. All gatherings involving large crowds *e.g.*, church attendance, conferences, festivals, and trade fairs, were prohibited to limit person-to-person contact. A surveillance system was put in place by the Ministry of Health to enable rapid detection and isolation of suspected cases ( Wangari *et al.,* 2021). Also, new protocols for the handling of diseased remains and conducting funerals were issued by the Ministry of Health ( Wangari *et al.,* 2021). Additionally, several other control measures including quarantine (isolation of infected persons), wearing of masks, and hand washing were put in place to prevent the spread of the disease. In fact, the government launched a nationwide campaign to educate the public on proper use of face masks and hand washing techniques.

The situation was rather different in Tanzania because a full lockdown was never imposed. However, Tanzanian schools were shut for over two months from April 2020. This was accompanied by limitations in research activities and gatherings. Restrictions were mainly left to the institutional level, with each institution, including schools, putting in place their own policies. These ranged from general hygiene and social distancing measures to the exclusion of entry for all visitors.

## The THRiVE project

Support and mentoring have been provided to scientists at various stages of their research careers through THRiVE, including graduate interns, Masters, doctoral and post-doctoral researchers. The focus of all the research funded by THRiVE has been on health, specifically in the areas of (a) infectious diseases/neglected tropical diseases; (b) maternal, neonatal and reproductive health, and (c) non-communicable diseases. A total of 70 research fellows have benefitted from supervision, teaching and mentoring in the project across the three East African countries. In this paper, we focus on four of these researchers: Trizah K. Milugo from Kenya, who was conducting research on the impact of invasive plant species on malaria mosquito ecology and bionomics ( Milugo *et al.,* 2021a; Milugo *et al.,* 2021b; Milugo *et al.,* 2021c.). Currently, the sub-Saharan African region carries a disproportionately high share of the global malaria burden, with many of the annual deaths preventable ( World Health Organization, 2021). Trizah's research not only contributes new knowledge to the science of mosquito bionomics, but the findings will also inform current and future efforts aimed at designing interventions that prevent and reduce malaria deaths in the region. Mary Vincent Mosha, who conducted a study on the ‘Double Burden of Malnutrition: Prevalence and Correlates of over and underweight among primary school children in Kilimanjaro, Tanzania’ ( Mosha *et al.*, 2021; Mosha *et al.*, 2022). Rune Philemon from Tanzania, with research focused on breastfeeding among HIV-positive women ( Philemon *et al.*, 2022), respectively and Eddie Wampande from Uganda, who conducted research on tuberculosis sub-lineages in Kampala ( Wampande *et al.*, 2019).

Each researcher, as a part of their commitment to sharing their research with the communities they worked with, planned a series of community engagement activities with schools in their research area. We describe these plans, and what was accomplished before the pandemic curtailed activities, in the next section.

## Plans for community engagement and involvement activities before the pandemic

In Kenya, Trizah began her high school-based engagement activity in mid-2019. The engagement activities were aimed at promoting science literacy among high school students in her research area. The CEI program included mentorship sessions, science in action demonstrations, and science-based conversations using creative writing and drawing, poetry, and drama. The mentorship session involved both open forum career talks and one-on-one dialogue with students by Trizah. Under the science-in-action demonstration, the students were given an opportunity to design a project that was focused on a disease of public health importance. Thereafter, an exhibition was organized to showcase different projects, and the winning students were awarded certificates. Similarly, students who participated in the science-based conversations were given the opportunity to present their work during an open day, where they put on a performance of their material which was acknowledged and appreciated by those who attended the event. Most of the presentations were focused on malaria transmission modalities, causes of malaria, its prevention and control. Western Kenya is a malaria-endemic region and thus, it was not surprising that most of the activities were focused on malaria ( Were *et al.,* 2019).

Mary, working in Tanzania, near Mount Kilimanjaro, also began her CEI activities in 2019. Working in a secondary school, she worked with students to design their project. The students designed a health promotion activity, which focused on teaching their younger peers about healthy eating and the importance of physical activity. The activity was divided into two sessions: 1) teaching primary school students: classroom session, pre and post assessment and handling of healthy plate stickers; 2) participating in physical activity *i.e..*, run for fun from school premises to the nearby playgrounds, thereafter all students (from primary and secondary) participated in different games.

In another secondary school in a different part of Tanzania, in 2019, Rune worked with students who were in a rural boarding school to design and pilot various modalities for teaching about the prevention of HIV. This was seen as a way to help determine what methods work better with young people when it comes to education on HIV and HIV prevention. A group of biology students was split into four groups, and each group had to produce a way they would use to teach their fellows about HIV and HIV prevention. The methods would then be compared in teaching other students within the school to see which method resulted in the most improvement in knowledge. The proposed methods among the four groups were a comic book, a play, an interactive website, and a leaflet. The plan was, upon completion of the tools for the different teaching methods, to form four different test groups of students, with each group having members from across all the classes at the school. The test groups would then do a pre-test to establish their baseline knowledge of HIV, after which each group would be taught using one of the methods developed. A post-test would then be used to rank the average improvement in knowledge in the different test groups.

In Uganda, Eddie, was unable to begin any CEI activities before the lockdown. However, in 2021 with the help of the school teachers, 30 students were traced to their homes, and the parents were verbally consented to have their children enrolled in the activity. Of these, 20 were willing to participate in the initial engagement through a virtual (Zoom) meeting where the different modes of CEI in schools were discussed, especially infectious diseases, such as tuberculosis. Students also shared their thoughts on why girls were fewer than boys in taking the science disciplines in Uganda. The students were asked to prioritize the types of CEI delivery approaches that could be adopted in the school to accurately convey the message about tuberculosis in the community. Three activities were prioritized: composition of a poem, song, and a play in order of increasing interest. With the help of the school teachers, the students co-created a poem, a song, and a play. These three activities were presented during a dissemination workshop where different stakeholders were invited (from other schools, and from the ministries of health and education). The students then agreed to form a health club in the school so that they could continue with the efforts of communicating health issues in the school

## Impact of school closures and COVID-19 restrictions on planned activities

In March of 2020, the government in Kenya immediately imposed a nationwide closure of all schools. This made it impossible for Trizah to continue with the engagement activities. She attempted to implement the pending activities remotely through digital platforms. The efforts however were not fruitful as the schools were located in rural areas and lacked the infrastructure to support access through the internet. Furthermore, when schools reopened in 2021, most learning institutions were not accepting visitors on their premises, and this further derailed the community engagement activities. Consequently, Trizah had to wait until 2022 to continue with the programme of activities. This situation came with associated costs. She had to purchase face masks for the students to wear and hire assistants to help with small group sessions to comply with the restrictions on group size.

The schools Mary was working with in Tanzania closed in April 2020. Mary had prepared for this eventuality and had collected parents' telephone numbers for all participating students so that she could plan activities while they were at home. Unfortunately, this posed challenges, since parents were not always at home, so they could not link their child with Mary on the telephone. After the situation changed in 2021, Mary was able to go back to the school and start working with a new nutrition class. In preparation for this, Mary made several visits to the school to meet the administrators and the head teacher to plan how she would ensure the safety of the students. She provided students with face masks and sanitizers during the sessions. A second activity was to develop a comic book for use in primary schools. Mary managed to invite a cartoonist to the school who spent three days with students to assist them in designing the comic book. This was completed successfully.

For Rune, school closures in Tanzania came when students were still sketching out their ideas and trying to see how they could implement them. Plans to continue online with the discussion could not be implemented because not only were there problems with connectivity in the rural area where the schools were situated, but not all students had access to a smartphone. The situation was further complicated by the government suspending all research activities, and Rune being called back to work to aid with the COVID-19 efforts. It took four months before schools fully opened in August 2020, and, even then, visitors were not allowed into the schools. The students were behind in their studies, and with national examinations imminent, their timetable did not allow for any extracurricular activities such as those planned. Rune finally managed to go back to the school just as the class he had been working with was graduating.

The situation in Uganda for school closures were the most difficult among the three countries. When schools shut in Uganda in March 2020, they then remained shut for two years. This made it impossible for Eddie to continue with the program of face-to-face activities with the students. Eddie, like Trizah, tried at first to engage with students using zoom, six months after the school closure had begun. When schools reopened, Eddie attempted to restart the engagement activities by holding a face-to-face meeting. Unfortunately, the school administration was not supportive of this plan because, as Rune found in Tanzania, the students were so behind in their studies that it was impossible to fit other activities into the timetable. After discussion with the school management team and the students, Eddie agreed to undertake the engagement activities at the weekend and during holidays.

## Results of the community engagement activities in the different sites

In Kenya, Trizah found that the partnership she had established around the community engagement activities with school students, their teachers, and faculty members from the university was valued by all the partners. A student evaluation conducted during and again after the engagement activities showed that the use of creative writing and drawing used to engage students was valued. The methods helped students to remember the information they had been told, specifically methods of malaria prevention and care (use of insecticide-treated mosquito nets, maintaining environmental cleanliness, and seeking medical advice whenever one shows malaria symptoms). The high school teachers, on the other hand, acquired knowledge and skills that they could apply to their teaching, including information on malaria as well as setting up of science-in-action demonstration practicals.

For Trizah, the engagement activity provided a platform to improve on her mentorship skills and to forge relationships with school teachers, university faculty members, and university student mentees. Additionally, she learnt more about local knowledge and beliefs surrounding disease prevention and control, especially malaria, from the projects showcased by the students. Involving university faculty members in the community engagement project was important because they helped with implementing the project and assisting with student projects.

Mary was successful in completing her engagement activities, partly because the schools in Tanzania were less affected by closures than in Kenya and Uganda. The cartoonist was successful in guiding the students to produce the comic book, which they did themselves under his supervision. The comic book was completed and published in June 2022. The comic book was launched in that month and has been distributed to primary schools in the area Mary was working.

Rune was unable to complete his planned activities, but this work laid the foundation for further work. Rune was able to use the ideas the students came up with to develop an interactive quiz aimed at teaching young people about HIV. He also developed a comic book. He plans to test these materials with school students soon and to work with them to adapt them for their use and the use of other students.

Despite the delays in implementing the final stages of his community engagement activities, Eddie was able to work with students to produce a song, poem, and play about tuberculosis. The students were keen to make wall drawings, but the school administration would not allow the school walls to be decorated in this way. A science health club was proposed by the students, and this was welcomed by the school management. Come 2023, the students who participated in the PE will establish the club.

## Lessons learnt

Early in the pandemic, there were hopes that some activities with the school students could continue using virtual means. Nowhere was this successful because of poor connectivity, as well as many of the poorer students lacking access to telephones and computers.

When schools reopened and physical public engagement meetings were possible, blending these activities with lessons was a big challenge because of the time lost studying during the pandemic; teachers were reluctant to make time for extracurricular activities.

On the positive side, engagement activities offered new ways for students to think about subjects which they found exciting and interesting. The students enjoyed doing different activities (making up songs, drawing comics, and taking part in quizzes), which were different from their usual mode of learning in school.

## Conclusion

We have provided a detailed discussion on the impact of the pandemic on community engagement activities in Kenya, Uganda, and Tanzania. As we show, despite the delays and disruptions caused by the lockdowns particularly in Kenya and Uganda, we were able to find ways to engage and share our research with the next generation of researchers. We agree with Tindana *et al.* (2020) that during the COVID-19 pandemic community engagement has been essential for building and, as they write `maintaining relations of trust between communities and research institutions’ (p. 7).

## Disclaimer

The views expressed in this article are those of the author(s). Publication in Open Research Africa does not imply endorsement by F1000 or our partners.

## Data Availability

No data are associated with this article.
