# Supplementary material for: Public engagement by early career researchers during the COVID-19 pandemic: case studies from East Africa
Source: Open Res Afr. 2023 Dec 15;6:4. Originally published 2023 Mar 20. [Version 2] doi: 10.12688/openresafrica.13897.2 (PMC11252639; doi:10.12688/openresafrica.13897.2)
Supplement: Peer review history

# Reviewer report: Reviewer response for version 2

**DOI:** 10.21956/openresafrica.15661.r31491

**Published:** 2024-07-25

**Authors:** Ana Margarida Sardo

**Affiliations:** 1 University of the West of England, Bristol, England, UK

This is a very interesting letter, pertinent and timely. We need more studies reporting on the researchers’ reflections, as these can be crucial in informing other researchers and future projects. Can I please congratulate the authors on preparing this piece.

 A few recommendations for what I think may strengthen the open letter:

 1. I would welcome more detail in the section “Plans for community engagement”. Whereas I acknowledge this is not a research paper, it would enrich the piece if the authors added a bit detail on the numbers of schools, classes and students engaged in each case-study.

 2. When referring to the schools, could you add the age range for each. For example, high school (11 to 16) or something along those lines.

 3. As I said before, I understand this is a letter and not a full research paper. However, it would greatly improve the paper if mor context was given around how the researchers’ reflections were collected. For example, were they interviewed? Or maybe a structured self-reflection system was in place?

 4. It’s lovely and very positive to hear that the students enjoyed doing different activities (page 6). Could you, very briefly, add how you know this?

 5. Can you create a table to clearly state: the CEI steps, the revised CEI steps, and any strategies/outcomes/lessons learnt for each.

 Overall, the writing is good and very clear. The letter is relevant, timely, and adds to the body of knowledge in this area.

Does the article adequately reference differing views and opinions?

Yes

Are all factual statements correct, and are statements and arguments made adequately supported by citations?

Yes

Is the Open Letter written in accessible language?

Yes

Where applicable, are recommendations and next steps explained clearly for others to follow?

Yes

Is the rationale for the Open Letter provided in sufficient detail?

Yes

Reviewer Expertise:

Science communication and public engagement

I confirm that I have read this submission and believe that I have an appropriate level of expertise to confirm that it is of an acceptable scientific standard.

# Reviewer report: Reviewer response for version 2

**DOI:** 10.21956/openresafrica.15661.r31495

**Published:** 2024-07-16

**Authors:** Shannon Casey

**Affiliations:** 1 University of Wisconsin-Madison, Madison, Wisconsin, USA

The manuscript is a revision of a paper addressing Community Engagement and Involvement (CEI) in three East African countries. The value of international research cannot be understated. Thank you for amassing these case studies from a career development (THRiVE) initiative.  Overall, the content has much value. Descriptions of (what we in clinical and translational research call) facilitators and barriers of pivoting community engagement mechanisms in the COVID era have value for others doing community-engaged work. 

 A few recommendations for what I perceive may strengthen the manuscript, though may be outside the scope of what the others wish to do. 

 1.)  Can you please clarify (a sentence or two?) whether each of the four cases is an ACTUAL research study (e.g., IRB, protocols, funders) or whether there was a mix of a few cases being research studies and others being community development in advance of/subsequent to research activity?  The reason this matters is that CEI is relevant at all stages (e.g., pre-research, during, and post-research). It was a little difficult to perceive exactly where in the research process that these CEI activities occurred. It was also difficult to determine what was a study of community engagement/dissemination itself (e.g., Eddie searching for how youth might contribute to dissemination of tuberculosis education) and what was a training opportunity for a researcher to learn what community engagement means (e.g., Mary's study of teaching primary school students about healthy eating and physical activity). Because people who train others on CEI and people who conduct CEI are somewhat different audiences (not exclusively!), then a small amount of clarification that both are included here will help the reader. 

 2.)  Reading through the Plans, Impact, and Results sections (thoughtfully laid out in response to previous reviewers), I was looking for more detail on ACTION STEPS that the four researchers took to maintain/pivot/rebuild connections. What I am able to understand is:  Trizah delayed activity and brought face masks. Mary called parents, revisited school, and provided face masks etc and invited a cartoonist to school. Rune went back to the school, and Eddie tried Zoom, attempted engagement but was denied by the school and moved participation to weekends. Somehow I'm guessing that you all did a lot more than what is described (email communications, phone calls, collaborative events, consulting with colleagues?). To me, that is the meat of the paper!  Facilitators to maintaining engagement (sourcing another outlet - parents; engaging administrators often; understanding school needs and problem-solving specific diverse and multiple solutions) and barriers to maintaining engagement (contextual factors like physical closure; individual researcher factors like work demands). See below... A list of those barriers and facilitators would be super useful for your reader, if it's possible. 

 3.  Would it be possible to create a table to accompany the manuscript that lists in bullet points any of the following that you would deem valuable: 

 a.) Planned CEI steps

 b.) Redirected CEI steps

 or c.)  Strategies for CEI and then indicating which ones were planned, versus which ones were reactions to stalled plans?  A table might be valuable so readers can get a quick synopsis of action steps and pivots if those action steps don't work for whatever reason. As we all know, schools pull out of research, community funding falls through, community priorities change... so a bit more focus on the actual steps to pivot would be valuable. We would all learn from that!

 4.  In the Plans for CEI before the pandemic, Eddie's work seemed to start after the pandemic started. Had the planning started before? And how did CEI planning happen if the community had not been engaged? Clarification might help the reader. 

 5. In the last sentence before the "Lessons Learnt" section (page 6 of 12), the acronym PE is used. 

 Overall, the writing is very good and the support literature is relevant, timely, and easily accessible.

Does the article adequately reference differing views and opinions?

Yes

Are all factual statements correct, and are statements and arguments made adequately supported by citations?

Yes

Is the Open Letter written in accessible language?

Yes

Where applicable, are recommendations and next steps explained clearly for others to follow?

Partly

Is the rationale for the Open Letter provided in sufficient detail?

Yes

Reviewer Expertise:

Clinical and translational researcher; methodologist; evaluator of clinical and translational research impact

I confirm that I have read this submission and believe that I have an appropriate level of expertise to confirm that it is of an acceptable scientific standard.

# Reviewer report: Reviewer response for version 2

**DOI:** 10.21956/openresafrica.15661.r30456

**Published:** 2023-12-23

**Authors:** Sandra Lopez-Verges

**Affiliations:** 1 Gorgas Memorial Institute of Health Studies, Panamá, Panama; 2 Sistema Nacional de Investigación de SENACYT, Panama, Panama

Thank you very much to the authors for the revision of the manuscript following the reviewers' comments. I have no new comments.

Does the article adequately reference differing views and opinions?

Partly

Are all factual statements correct, and are statements and arguments made adequately supported by citations?

Yes

Is the Open Letter written in accessible language?

Yes

Where applicable, are recommendations and next steps explained clearly for others to follow?

Partly

Is the rationale for the Open Letter provided in sufficient detail?

Yes

Reviewer Expertise:

Virology, Immunology, COVID19 response, COVID19 impact on ECRs, science diplomacy and communication.

I confirm that I have read this submission and believe that I have an appropriate level of expertise to confirm that it is of an acceptable scientific standard.

# Reviewer report: Reviewer response for version 1

**DOI:** 10.21956/openresafrica.15052.r30218

**Published:** 2023-10-12

**Authors:** Sandra Lopez-Verges

**Affiliations:** 1 Gorgas Memorial Institute of Health Studies, Panamá, Panama; 2 Sistema Nacional de Investigación de SENACYT, Panama, Panama

This Open letter focuses on the important topic of the difficulties to develop Community engagement and involvement (CEI) for more participative research, when specific conditions, like the COVID-19 pandemic, do not allow for in-person interactions and activities between researchers and the community. The letter is well written, clear, based in four case examples, which are presented with sufficient details, that allowed discussion of the issues which arise during these times in settings where completely virtual interactions were not possible. This letter is relevant to researchers that would like to incorporate community engagement in their projects in general and in these specific settings in particular.

**Minor comments:**

1. The title could be without repetition of the region: East Africa does not need to be repeated, it needs to be stated only once, authors should choose which position they prefer to choose.
2. It is not clear what the brackets mean in the first paragraph of the introduction; it confuses the reader and makes the phrases less clear.
3. Please check the writing to avoid repetition of words or concepts in the text itself. For example, in the Introduction on page 3 of 9, the phrase “That project was a collaborative research capacity building project…” could be changed to “This was a collaborative research capacity building project…” to avoid the repetition of “project” in the same phrase. Similarly, try to avoid repetitions on two subsequent phrases using pronouns or synonyms. For example, on page 5 of 9, “The comic book was completed and published in June 2022. The comic book was launched in that month and has been distributed to primary schools in the area Mary was working” could become “The comic book was completed and published in June 2022. It was then distributed to primary schools in the area Mary was working”. This will improve the text.
4. In the introduction on page 3 of 9, please cite the articles associated with findings on engaging populations on topics different than COVID during the pandemic, as you cited in the phrases before the ones associated with COVID-19 research. This is especially important as you state that the work presented and discussed in this open letter contributes to this group of work.
5. In the section about the 3 countries’ measures on page 3 of 9, there is a citation only for Kenya’s measures, if possible, try to include publications for Uganda and Tanzania. Similarly, Kenya’s measures are described in detail, please try to resume the information as done for the other two countries, keeping the main points, particularly on school measures, as the CEI activities described in the letter were planned for schools.
6. In the section on the THRive project on page 4 of 9, the part of Trizah’s research is described more in detail than for the other 3 researchers, please keep it as short as for the others.
7. In the Lessons learnt section on page 6 of 9, the two first paragraphs are a resume of what was described before, the text could be strengthened if each observation were redacted as generalized lessons (e.g. virtual activities were not successful), and should be compared with other studies in the THRive project, previous studies or observations in the same region or in other regions of the world with similar issues. This will give a higher impact to the letter.
8. After the paragraph on the positive side of these cases, a small paragraph on perspectives or actions for having a less negative impact on CEI activities should be discussed, as some of the examples, like Eric and Rune, did have to stop their program losing some of the initial participants. What could be done so this does not happen if another extreme situation happens? This could allow the letter to keep the main message for future strait to the reader.

Does the article adequately reference differing views and opinions?

Partly

Are all factual statements correct, and are statements and arguments made adequately supported by citations?

Yes

Is the Open Letter written in accessible language?

Yes

Where applicable, are recommendations and next steps explained clearly for others to follow?

Partly

Is the rationale for the Open Letter provided in sufficient detail?

Yes

Reviewer Expertise:

Virology, Immunology, COVID19 response, COVID19 impact on ECRs, science diplomacy and communication.

I confirm that I have read this submission and believe that I have an appropriate level of expertise to confirm that it is of an acceptable scientific standard, however I have significant reservations, as outlined above.

# Reviewer report: Reviewer response for version 1

**DOI:** 10.21956/openresafrica.15052.r29579

**Published:** 2023-04-13

**Authors:** Doreen Tembo

**Affiliations:** 1 School for Healthcare Enterprise and Innovation, Faculty of Medicine, University of Southampton, Southampton, UK

This is a well written and summarised account of the community engagement efforts of three researchers working in the THRiVE research project. The article focuses on lessons learnt of carrying out community engagement in the face of lock-downs during the Coronavirus pandemic. The article is a valuable addition to learning in this area.

 I only have a few minor points including:

1. It is unclear what the parenthesis [] denote in the introductory paragraph.
2. The authors should give a clear definition of what they define as ‘Community Engagement and Involvement’ in this article. The article appears to mostly focus on science communication?
3. Better linkages could be made between the role of the engagement activities and the Thrive research project i.e. what impact did the activities play in relation to the Thrive project, if any?
4. The lessons learned could highlight the importance of contingency planning and flexibility as well as relationship building with key stakeholders/gatekeepers (e.g. school staff and parents).

Does the article adequately reference differing views and opinions?

Yes

Are all factual statements correct, and are statements and arguments made adequately supported by citations?

Yes

Is the Open Letter written in accessible language?

Yes

Where applicable, are recommendations and next steps explained clearly for others to follow?

Partly

Is the rationale for the Open Letter provided in sufficient detail?

Yes

Reviewer Expertise:

public health research, health policy research , health systems research, patient and public involvement and engagement, community engagement and involvement

I confirm that I have read this submission and believe that I have an appropriate level of expertise to confirm that it is of an acceptable scientific standard, however I have significant reservations, as outlined above.

# Response

**Published:** 2023-11-06

**Authors:** Trizah Koyi

**Affiliations:** icipe, Kenya

1.The parenthesis has been removed 2. The author have added the following statement to differentiate between science communication and CEI “Community engagement and involvement refers to a two-way interaction between the scientist and the community with scientist sharing information and also learning from the community; it differs from science communication which is a one-way form of interaction where information flows only from scientist to the public”. 3.  The activities were a part of the overall engagement activities undertaken by each THRIVE fellow – as such these activities did contribute to learning across the project on researcher engagement.  We have added this wording on page 6/7: `As part of their skills development, each fellow was supported to develop their skills in community engagement.  The learning from these activities was shared as part of annual meetings which brought together the fellows, their supervisors and mentors.’ 4. This aspect has been added. Thank you
